# The association between denture self-satisfaction rates and OHRQoL - a follow-up study

**DOI:** 10.1186/s12903-020-01119-1

**Published:** 2020-05-12

**Authors:** Chia-Jen Teng, Sheng-Che Lin, Jen-Hao Chen, Yi Chen, Hsiao-Ching Kuo, Pei-Shan Ho

**Affiliations:** 1 Public Health Bureau, Tainan City Government, Tainan, Taiwan; 2 School of Dentistry, College of Dental Medicine, Kaohsiung Medical University, Kaohsiung, Taiwan; 3 Tainan Municipal AN - NAN Hospital - China Medical University, Tainan, Taiwan; 4 Dental Department, Kaohsiung Municipal Hsaio-Kang, Kaohsiung, Taiwan; 5 Department of Prosthodontics, Kaohsiung Medical University, Kaohsiung, Taiwan; 6 Department of Pharmacy, Kaohsiung Chang Gung Memorial Hospital, Kaohsiung, Taiwan; 7 Department of Oral Hygiene, College of Dental Medicine, Kaohsiung Medical University, Kaohsiung, Taiwan; 8 Division of Medical Statistics and Bioinformatics, Department of Medical Research, Kaohsiung Medical University Hospital, Kaohsiung Medical University, Kaohsiung, Taiwan

**Keywords:** Oral health, Elderly, Satisfaction, OHRQoL, OHIP-7T

## Abstract

**Backgroud:**

The objectives of this study were to try to identify the key dimension in satisfaction from the combination of satisfaction clusters, and its effect on the change of OHRQoL(Oral Health-related Quality of Life) of elderly denture users.

**Methods:**

This follow-up study was conducted in subjects aged 65 years and over. All participants (*n* = 2128) completed questionnaires before and approximately 6 months after receiving complete denture. Information obtained by questionnaire included demographic characteristics, patients’ self-satisfaction rate and OHRQoL. The 6 satisfaction dimensions (including speaking, stability, esthetic, chewing, doctor and general dimensions) were classified as 5 cluster groups, which is the group of **n**ot at **a**ll **s**atisfied in all dimensions (NAS); only **s**atisfied with **d**octor and **g**eneral dimensions(SDG); **m**oderate **s**atisfaction group(MS); **q**uite **s**atisfied group(QS); the **h**ighly **s**atisfied group(HS) by an analysis of PCA (Principle component analysis) and CA (cluster analysis). Multiple linear regression was adapted to estimate the association between satisfaction and the responsiveness of OHIP-7T (Oral Health Impact Profile).

**Results:**

When compared to the cluster “NAS”, the greatest improvement of OHRQoL after treatment was found in the group “HS” (β = 7.31(6.26–8.36), followed by group “QS” (β = 4.71(3.54–5.87)), group “MS” (β = 4.33(2.92–5.74)) and group “SDG” (β = 3.25(2.10–4.41)). An increasing trend was detected in patient-rating satisfaction and OHRQoL. The satisfaction cluster group is an important factor of OHRQoL after adjusting for other confounders.

**Conclusion:**

Psychological-related aspects is the greatest impacting dimension on OHRQoL among denture wearers in Taiwan elderly. Better communication from the dental professional team with denture patients would improve their OHRQoL.

## Background

Patient-report outcome (PROs) provide practice guides for clinical treatment. Despite declining edentulism and increasing implant treatment, the need for complete denture treatment will remain substantial in the future [1–4]. For most edentulous patients in Taiwan, complete denture will be the first treatment option [5]. The growing elderly population worldwide and the extended edentulism status have increased the need for successful denture treatment. Denture treatment is a time-consuming process, the follow-up adjustment of denture fitting and tissue adaptation to get a satisfactory outcome is important. It has been found that psychological factors may play an important role in those patients who experience difficulty in adapting to new dentures [6–8]. The function, social and psychological impact of edentulous status have been discussed in many previous studies [1, 4, 6, 9–12]. PROs allow for the straight quantification in patients’ opinion of distinct aspects of denture intervention.

During clinical trials, patient-ratings in treatment satisfaction are often assessed along with quality of life [13–15]. It has been suggested that self-ratings of satisfaction may be more sensitive to change than quality of life, particularly for comparisons of palliative treatment for chronic medical conditions [16]. Self-rating satisfaction will vary with an individual’s preferences, expectations and with the quality of the information given by the physician [14, 17].

Patient’s satisfaction [1, 15, 16, 18] and OHRQoL [19–24] are the most common factors to consider in patient-centered analysis of prostheses treatment. The OHIP is a reliable and valid instrument suitable for assessment of OHRQoL in cross-sectional and longitudinal studies [1, 3, 4, 25–27]. A number of studies have evidenced the correlation between patient satisfaction and quality of life. In Chen’s study, it was suggested that dentist-patient communication and denture quality are correlated with the patient’s satisfaction. Joselyn’s study also showed a positive association between dental satisfaction and OHRQoL. Kao’s paper [4] suggested that denture treatment is associated with responsiveness of OHRQoL With the complex correlation between various dimensions of satisfaction and OHRQoL, we need a more comprehensive assessment of satisfaction patterns [4, 5].

The correlation between expectation and satisfaction after complete denture treatment has been assessed in some previous studies [28–30]. However, this satisfaction of treatment is multi-dimensional concept. There is a discordant conclusion about the relationship between denture satisfaction and OHRQoL [5, 7, 31–33]. The objectives of this study were to try to identify the key dimension in satisfaction from the combination of satisfaction clusters, and its effect on the change of OHRQoL of elderly denture users.

## Methods

### Study subject

This follow-up study was conducted in accordance with the welfare plan: ‘Dentures for the Elderly through Public Funding’ conducted by the Tainan city government, Taiwan. This plan provides a complete set of removable dentures free of charge for elderly city residents who are aged 65 years and over. While price restrictions are imposed in the procurement of the removable denture, no restriction is imposed on manufacture of the denture. All 2128 participants completed the first questionnaire of their OHRQoL before device denture fitting and the second questionnaire about their self-rating satisfaction and OHRQoL were completed approximately 6 months after complete denture use. All questionnaires were collected by the staff of the Health Bureau of Tainan city government. And all the interviewers have accepted standard training. This study was approved by the Institutional Review Board (Human Experiment and Ethics Committee, Kaohsiung Medical University Hospital, KMUH-IRB-EXEMPT-20140056).

### Questionnaire

Information obtained by questionnaire included demographic characteristics (age group, gender, economic status), questions regarding patient-satisfaction with their new complete denture, and OHRQoL The economic status were collected by participants self-assessing as “very well”, “well”, “poor”, “very poor”. This study is a community-based health program evaluation. To consider the practicality and efficiency of the government health program evaluation, the shortened OHIP-7T [34] was used to assess OHRQoL.

Six dimensions of satisfaction with complete denture were assessed in the questionnaire, which were speaking, stability, esthetic, chewing, doctor and general. Each question was evaluated on a Likert 5-point scale, from ‘excellent’ (score = 5), ‘good’ (score = 4), ‘fair’ (score = 3), ‘poor’ (score = 2) or ‘very poor’ (score = 1) to ‘very poor’ (score = 1). Higher satisfaction was indicated by a higher score. OHRQoL was measured using the Taiwanese (Chinese) version of OHIP-7T, which was developed and validated by Kuo et.al [34]. In community-based studies, it is very important to collect information efficiently in limited time. OHIP-7T composed one question in every domain of the 7 dimensions. For each OHIP item, patients were asked how frequently they had experienced the oral health impact of that item in the 3 months prior to treatment and at evaluation 6 months post-treatment (after completion of new complete denture). Responses of OHIP-7T questions were also made on a 5-point scale, which indicated if the problem had been experienced ‘very often’ (score = 4), ‘fairly often’ (score = 3), ‘occasionally’ (score = 2), ‘hardly ever’ (score = 1) or ‘never’ (score = 0). There are seven conceptual dimensions (function limitation, psychological, pain discomfort, physical disability, psychological disability, social disability, and handicap) in OHIP-7T. The total OHIP-7T score was a summation of each item score.

Since only one question was evaluated in each conceptual dimension, all of them were dichotomized into “very often/ fairly often” as impact; and “occasionally/hardly ever/never” as no impact when considering individual item effect.

### Cluster of satisfaction

Cluster analysis is a class technique that classified cases into groups that are relatively heterogenous between each other [35] .Satisfaction of denture treatment highlight multidimensional assessment, it is important to classify responses into relative homogenous and meaningful subgroups. The subgroups may provide a more comprehensive opinion for assessing psychosocial factors. Hierarchical cluster combines cases into homogeneous cluster by merging them together one at a time in a series of sequential steps [36]. Principle component analysis (PCA) and cluster analysis (CA) are two commonly used statistical approaches to provide information about existing patterns with the population. PCA uses the correlation matrix of satisfaction dimensions to identify common patterns of satisfaction within the data in order to account for the largest amount of variation of satisfaction. CA groups individuals with similar satisfaction patterns into mutually exclusive categories according to the mean of satisfaction variable. Several CA algorithms exist with K-means being popular in CA research because it can handle a large number of input variables efficiently.

The six satisfaction dimension scores were entered into Wald’s hierarchical cluster analysis. Examination of the agglomeration schedule and, subsequent group mean profile for a range of cluster solutions strongly suggested a five cluster solution. The five cluster group is the group of **n**ot at **a**ll **s**atisfied in all dimensions, named “NAS” group; only **s**atisfied with **d**octor and **g**eneral dimensions, named “SDG”; the third group showed **m**oderate **s**atisfaction in the five dimensions, named “MS”, and the 4th group is **q**uite **s**atisfied, named “QS”; the last group is very **h**ighly **s**atisfied with all dimensions, named “HS”.

### Statistics methods

Participant characteristics across different satisfaction groups were explored using Chi-square analysis. The responsiveness of each OHIP dimension was evaluated by McNemar’s statistics, which is based on the total number of the discordant at pre- and post- treatment.

Cohen’s standardized effect size (ES) [37], was computed to evaluate the responsiveness of different measurements [4]. ES could be considered as several levels of clinical meaningfulness (small: 0.2 ≤ ES < 0.5; moderate: 0.5 ≤ ES < 0.8; large: 0.8 ≤ ES). The responsiveness of OHIP-7T was compared among the five satisfaction groups. The comparison of different satisfaction groups in OHIP improvement with respect to the complete denture intervention was assessed by one-way analysis of variance (ANOVA). Multiple linear regression analysis was adapted to estimate the association between patient satisfaction and the responsiveness of OHIP-7T, while adjusting for pretreatment score, gender, age, education level, economic status, and personal habits (including cigarette smoking, alcohol drinking and areca quid chewing), the experience of complete denture use before, perceived oral and general health. Trend test of different satisfaction groups was performed by using the exposure measurement as continuous predictors in multiple linear regression. The effect of each OHIP dimension responsiveness was presented by Mantel-Haenszel estimator which is used to obtain an estimate odds ratio(OR) and 95% CI [38].

The statistical analyses were carried out by JMP12.1.0 (SAS Institute Inc., Cary, NC, USA).

## Results

**Fig. 1:**
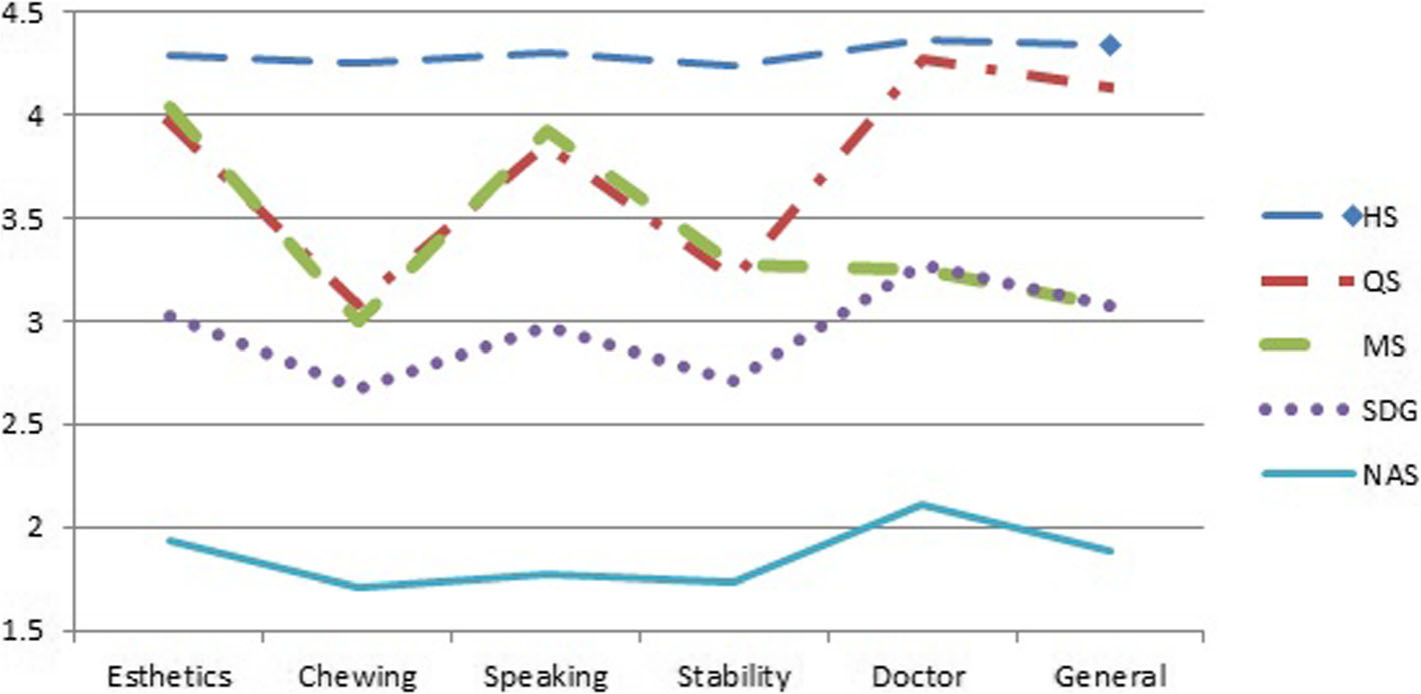
The dimension patterns of satisfaction cluster groups

In Table 1, a strong correlation (*p* value < 0.001 in all pairs) was found among the six different satisfaction dimensions. The five cluster solution is determined by cluster analysis. The means and standard deviation of six satisfaction dimensions across clusters demonstrated that the identified clusters had varied patterns of the six satisfaction dimensions in Fig. 1. The cluster “NAS” was the lowest satisfaction score at every dimension, and cluster “SDG” had a similar pattern, but a slightly higher satisfied score. A lower satisfaction pattern was found in the dimension of chewing ability, stability, doctor and general in cluster “MS”. And only dissatisfaction of chewing ability and stability was found in cluster “QS”. In cluster “HS”, a higher satisfaction score was found in six dimensions of denture use. The basic demographics and personal habits of the five clusters are shown in Table 2. A higher proportion of males was found in clusters “NAS”(51.81%), “SDG”(48.92%) and “MS”(55.68%), but the diversity did not reach a statistically significant difference. The education level, smoking cigarettes, areca quid chewing and alcohol drinking were similar in the five clusters. However, a lower economic status (11.56%) was found in cluster “HS”, which reached a statistically significant difference.

**Table 1:** Correlations between different dimensions of satisfaction

	Chewing	Speaking	Stability	Doctor	General
Esthetics	0.73	0.83	0.73	0.76	0.80
Chewing		0.75	0.83	0.66	0.77
Speaking			0.76	0.76	0.81
Stability				0.67	0.75
Doctor					0.83

*The P value of all pair correlation is < 0.001

**Table 2:** The demographic variables and personal habits in the satisfaction groups

	Total		Satisfaction clusters					
	N	%	“NAS”	“SDG”	“MS”	“QS”	“HS”	P value
			83	278	88	261	1418	
**Gender**								
Male	984	46.24	43(51.81)	136(48.92)	49(55.68)	107(41.00)	649(45.77)	0.0913
Female	1144	53.76	40(48.19)	142(51.08)	39(44.32)	154(59.00)	769(54.23)	
**The experience of denture use before**								
No	545	25.95	21(25.61)	77(27.80)	19(21.59)	80(31.13)	348(24.93)	0.2304
Yes	1555	74.05	61(74.39)	200(72.20)	69(78.41)	177(68.87)	1048(75.07)	
**Education level**								
Illiterate or under Elementary school	694	32.61	32(38.55)	100(35.97)	28(31.82)	100(38.31)	434(30.61)	0.1513
Elementary school	307	14.43	11(13.25)	41(14.75)	9(10.23)	34(13.03)	212(14.95)	
Junior high school	758	35.62	27(35.53)	84(30.22)	40(45.45)	83(31.80)	524(36.95)	
Senior high school	165	7.75	9(10.84)	19(6.83)	5(5.68)	22(8.43)	110(7.76)	
University above	204	9.59	4(4.82)	34(12.23)	6(6.82)	22(8.43)	138(9.73)	
**Economic status**								
Very well	673	31.63	27(32.53)	88(31.65)	25(28.41)	75(28.74)	458(32.30)	< 0.001
Well	1144	53.76	39(46.99)	136(48.92)	35(39.77)	138(52.87)	796(56.14)	
Poor	289	13.58	16(19.28)	47(16.91)	26(29.55)	43(16.48)	157(11.07)	
Very poor	22	1.03	1(1.20)	7(2.52)	2(2.27)	5(1.92)	7(0.49)	
**Cigarette smoking**								0.3809
No	1831	86.04	69(83.13)	245(88.13)	75(85.23)	216(82.76)	1226(86.46)	
Yes	297	13.96	14(16.87)	33(11.87)	13(14.77)	45(17.24)	192(13.54)	
**Alcohol drinking**								
No	1963	92.25	78(93.98)	258(92.81)	83(94.32)	235(90.04)	1309(92.31)	0.5945
Yes	165	7.75	5(6.02)	20(7.19)	5(5.68)	26(9.96)	109(7.69)	
**Areca quid chewing**								
No	2031	95.44	81(97.59)	269(96.76)	81(92.05)	244(93.49)	1356(95.63)	0.1506
Yes	97	4.56	2(2.41)	9(3.24)	7(7.95)	17(6.51)	62(4.37)	

The responsiveness of seven conceptual dimensions in OHIP-7T was compared among the five satisfaction groups. The mean of OHIP-7T before treatment, 6 months after treatment and observed difference is shown in Table 3. It shows that there was almost no difference in OHIP-7T before and after treatment in cluster “NAS” (ES = 0.12), which means that only 12% improved in OHIP-7T after denture treatment. And an increasing impact improved level was found in cluster “SDG” to “HS” by ascending sequence.

**Table 3:** Responsiveness of overall OHIP-7T in edentulous elderly with complete denture satisfaction groups

		Pre-treatment				Post-treatment				Difference				^a^ES
Group	Number	Mean	SD	0.0687		Mean	SD	< 0.001		Mean	SD	< 0.001		
NAS	83	12.36	6.25			11.61	9.18			0.75	10.04			0.12
SDG	278	13.64	5.92			8.62	6.65			5.02	8.38	NAS < SDG	SDG < HS	0.85
AS	88	12.84	5.81			7.25	5.47			5.59	7.73	NAS < AS	AS<HS	0.96
QS	261	13.12	6.01			6.62	5.02			6.50	8.04	NAS < QS	QS < HS	1.08
HS	1418	12.56	6.05			3.76	3.92			8.80	7.15	NAS < HS		1.46

^a^*ES*: effect size

The related factors affecting the responsiveness of overall OHIP-7T are presented in Table 4. Since there are some diversity existing among demographic variables, we will use statistical model to adjust these confounders. The satisfaction group is an important affecting factor after adjusting for demographic variables, personal habits, the experience of denture use, perceived oral and general health and pretreatment OHIP-7T score. To control the effect of confounding factors in improvement of OHRQoL, the statistical models were performed in Table 4 and Table 5. In Table 4, the regression coefficience in group “HS” was 7.31, which means that OHRQoL improvement in group of “HS” was 7.31 more than in in group of “NAS” after denture treatment. When controlling other confounders in model, the greatest improvement of OHRQoL after denture treatment was found in group “HS”(β = 7.31(95%CI: 6.26–8.36) compared to satisfaction group “NAS”, followed by group “QS” (β = 4.71(95%CI: 3.54–5.87)), group “MS”(β = 4.33(95%CI: 2.92–5.74)) and group “SDG”(β = 3.25(95%CI: 2.10–4.41)). A statistically significant increasing pattern was detected in satisfaction and OHRQoL from Table 4. The affected OHRQoL dimension levels of improvement between pre- and post-treatment at the five satisfaction cluster groups is presented in Table 5. The most improved OHIP dimension was observed at “psychological discomfort” in every cluster group. In group “NAS”, the other significantly improved OHIP dimensions were “handicapped”(OR = 2.18(95%CI: 1.06, 4.45)) and “function limitation” (OR=1.93(95%CI:1.01, 3.68)), which means that the subject with impact OHIP dimensions of “handicapped” and “function limitation” in pre-treatment are 2.18 and 1.93 times of the chance for having got improved after denture treatment separately. In group “HS”, with the exception of “psychological discomfort” (OR = 29.32(95%CI: 20.51, 43.56)), the dimension of “function limitation” (OR = 29.89(95%CI: 20.51.43.56)) is also an important improvement dimension.

**Table 4:** The factors related to responsiveness in OHIP for Taiwan elderly with new denture treatment

Variable		β	95%CI	P value
**Satisfaction group**	NAS			
	SDG	3.25	(2.10,4.41)	<.0001
	AS	4.33	(2.92,5.74)	<.0001
	QS	4.71	(3.54,5.87)	<.0001
	HS	7.31	(6.26,8.36)	<.0001
			P for trend< 0.001^a^	
**AGE**		−0.04	(−0.07,-0.02)	0.0024
**Gender**	Male			
	Female	−0.25	(−0.74,0.23)	0.3037
**Cigarette smoking**	No			
	Yes	0.30	(−0.44,1.04)	0.4319
**Alcohol drinking**	No			
	Yes	−0.09	(−1.04,0.86)	0.8562
**Areca quid chewing**	No			
	Yes	−0.65	(−1.85,0.55)	0.2906
**Education level**	Illiterate or literate			
	Elementary school	−0.14	(− 0.78,0.51)	0.6824
	Junior high school	−0.48	(−1.00,0.04)	0.0711
	Senior high school	−0.19	(−1.03,0.65)	0.6609
	above university	−0.85	(−1.64,-0.05)	0.0361
**Economic status**	Very well			
	Well	−0.64	(−1.10,-0.19)	0.0054
	Poor or very poor	−1.18	(−1.83,-0.53)	0.0004
**The experience of**	No			
**denture used before**	Yes	0.10	(−0.37,0.56)	0.6817
**Pre-treatrment OHIP-7 T**		0.98	(0.95,1.02)	<.0001
**Perceived oral health**		1.64	(1.23,2.05)	<.0001
**Perceived general health**		0.67	(0.32,1.02)	0.0002
				R^2^ = 64.90

^a^Trend test of different satisfaction group were performed by using the exposure measurement as continuous predictors in multiple linear regression

**Table 5:** The comparison of the five satisfaction groups in responsiveness of the seven conceptual dimensions OHIP-7 T after denture treatment

	Post-treatment															
Pre-treatment	“NAS”			“SDG”			“MS”				“QS”			“HS”		
**Function limitation**	No impact	Impact	OR^a^	No impact	Impact	OR^a^	No impact	Impact	OR^a^		No impact	Impact	OR^a^	No impact	Impact	OR^a^
No impact	18	14	1.93	58	26	4.88	19	9	5.56		71	18	7.67	493	28	29.89
Impact	27	24	(1.01, 3.68)	127	67	(3.20, 7.45)	50	10	(2.73, 11.30)		138	34	(4.69, 12.53)	837	60	(20.51, 43.56)
**Physical Pain**																
No impact	7	13	1.69	21	24	4.21	11	6	5.00		31	26	4.23	292	50	18.72
Impact	22	41	(0.85, 3.36)	101	132	(2.70, 6.57)	30	41	(2.08,12.01)		110	94	(2.76, 6.49)	936	140	(14.08 24.88)
**Psychological discomfort**																
No impact	16	9	3.22	52	16	8.25	20	6	7.00		52	17	8.29	401	31	29.32
Impact	29	29	(1.53, 6.81)	132	78	(4.91, 13.86)	42	20	(2.98, 16.47)		141	51	(5.01, 13.72)	909	77	(20.50, 41.94)
**Physical disability**																
No impact	10	14	1.43	28	25	3.08	7	12	1.83		23	32	2.75	231	109	6.91
Impact	20	39	(0.72, 2.83)	77	148	(1.96, 4.84)	22	47	(0.91, 3.70)		88	118	(1.83, 4.12)	753	325	(5.65, 8.45)
**Psychological disability**																
No impact	32	12	1.83	95	30	3.37	35	8	4.75		108	19	6.32	716	25	25.72
Impact	22	17	(0.91,3.70)	101	52	(2.24, 5.06)	38	7	(2.22, 10.18)		120	14	(3.89, 10.25)	643	34	(17.25, 38.35)
**Social disability**																
No impact	38	15	1.07	112	41	2.10	38	12	2.67		128	18	5.78	814	30	18.23
Impact	16	14	(0.53, 2.16)	86	39	(1.45, 3.04)	32	6	(1.37, 5.18)		104	11	(3.50, 9.53)	547	27	(12.63, 26.33)
**Handicap**																
No impact	29	11	2.18	87	34	3.21	31	9	4.56		114	15	7.80	683	25	27.04
Impact	24	19	(1.07, 4.45)	109	48	(2.18,4.71)	41	7	(2.21, 9.37)		117	15	(4.56, 13.35)	676	34	(18.14, 40.31)

^a^ The estimated Odds Ratios and 95%CI was calculated by Mantel-Haenzel estimator

## Discussion

The use of clinical measures only to assess the oral health of individuals has been criticized because they fail to consider functional and psychosocial aspects of health and do not adequately reflect the function, concerns and perceived needs of individuals [20–23, 39]. There is little evidence supporting association between patient-reported QoL and satisfaction [40, 41]. But, the collinearity of distinct satisfaction dimensions would be more complex when evaluating the relation between self-rated satisfaction and OHRQoL.

The satisfaction group is an important affecting factor after adjusting for demographic variables, personal habits, the experience of denture use, perceived oral and general health and pretreatment OHIP-7T score. Our paper is the first study to evaluate the multi-dimension cluster satisfaction patterns related to the OHRQoL, which provide a concept to deal with the collinearity of distinct satisfaction dimensions. We modified the analyzing method in each dimension of satisfaction used in previous studious. It was found that the responsiveness of OHIP-7T is acceptable in our study. There are several important findings in this study. The first is that the major satisfaction dimensions affecting OHRQoL are the dimensions of doctor and general. Also the OHIP improved pattern is strongly associated with satisfaction groups and also show a dose-response relationship. The second finding is that “psychological discomfort” is the most improved dimension of OHIP in every satisfaction group, and “function limitation” also showed great improvement in the “HS” satisfaction group. The third important finding is that satisfaction associated with “chewing ability” and “stability” are the major parts affecting overall OHRQoL. One of the advantages of using patient satisfaction as a treatment outcome is its simplicity and comprehensibility in a clinical environment [5]. From edentureous subject’s opinions, it is expected that new denture fit and function should be equal to or even better than their natural teeth [5]. However, the status of resorbed ridges, collapsed muscles and other physical changes would be the important baseline considerations. It should be an important part of dentist-patient communication before denture treatment. During the process of denture treatment, satisfaction can provide an insight into complete denture wearers’ physiological and psychological capacities [33, 42]. In our study, approximately 33% of denture wearers was dissatisfied with their denture, which is similar to previous studies [31, 43, 44]. Among all the dissatisfied wearers, two thirds showed dissatisfaction to their doctor and general dimensions. Chen’s study [5] showed that more than 30% of elderly people have experienced difficulty in adapting to their prostheses. Dentist-patient communication might play a key role in patients satisfaction regarding denture-wearing episodes, after-care concerns, and instructions related to nutrition, speech, nocturnal wear and denture hygiene [1, 2, 8, 45]. General health, age, gender, personality traits, experience with previous dentures and patient expectations regarding medical care were factors relating to the satisfaction of denture [29, 45–49]. The “psychological discomfort” is the most improved OHIP dimension, and this pattern is found in every satisfaction group. Although the improved impact in every satisfaction group varies. The dimension of “psychological discomfort” is the reflection of psychological capacities, which demonstrated the importance of dentist-patient relationship [5, 44, 50]. Denture wear not only comprised of good fabrication quality, but also denture-wearing episodes and after care concerns, individual follow-up schedules, and instructions related to daily wear. It is important to gain a deeper comprehension of patients’ psychosomatic phenomena, hence, more extensive, clinic and patient-based research should be carried out to gain more knowledge about patients’ expectations and final evaluation of complete denture treatment.

The function aspect of complete denture is also an important factor relating to OHRQoL. Michaud’s study found that a person’s perceived chewing ability and oral condition are the most important factors affecting OHRQoL [14]. The “chewing ability” and “stability” are related to eating and speaking capacity, which are the most important oral functions. The results of our study are in agreement with those of Awad and Feine [51], who demonstrated that the functional aspects (chewing and speaking ability) significantly affect the rating of satisfaction, and it also found this function aspect as having a critical impact on OHRQoL.

The items related to eating were the functions that most participants reported to be positively affected. This was an expected finding, as teeth are directly involved in chewing and biting, and thus enjoyment of eating. In this study, we found that both the satisfaction in function and psychosocial aspects of denture wearers significantly affect the OHRQoL. However, the dissatisfaction in doctor and general aspects had a higher impact on OHRQoL than in chewing capacity and stability of denture. Previous studies [51, 52] found that the ability of chewing is the most important factor in denture satisfaction. But in Tuker’s paper of 2009, the association between subjective reported satisfaction with the objective measure of denture quality such as retention and stability are often statistically insignificant [53]. In 2018, Luo’s study [54] realized that the denture wearers expected the attention and concern of their suffering from dentistry professional groups, including understanding their expectation of the denture treatment, their clarity of explanation about the treatment and follow-up adjustments. Further study is needed to examine the insight of the patient’s perception after denture treatment.

There are some limitations in our study. First, we did not encounter any discrepancies between outcome measures due to denture material, since the material and funding for complete sets of removable denture are standardized for all patients. Second, it is in terms of generalizability of these results. It is not known if similar findings would prove comparable if the OHRQoL tool was used with younger denture wearers. Third, our study proved that patient-based measures are associated with improvement of OHRQoL. But, the effect of expert-based measures on denture satisfaction and OHRQL improvement still needs further study in the future.

## Conclusion

In this study, we found that the satisfaction of doctor and general is the main dimension affecting improvement of OHIQoL after denture treatment. And it is also found that the importance of psychological aspects have greater impact than physical aspects in OHIP. Among denture wearers in Taiwan elderly, psychological value is the greatest impacting dimension on OHRQoL. Additional efforts should be made to improve satisfaction of patients with their dentures through development of communication among dental professional teams, and to improve the OHRQoL, which is one of the most important factors with denture wearers. Also, we expect that the shorter OHIP-7T will be a useful alternative instrument when time and resources are limited.

## Data Availability

The data sets used and/or analyzed during the current study are available from the corresponding author upon reasonable request.

## Abbreviations

OHRQoL: Oral health-related quality of life
PCA: Principle component analysis
CA: Cluster analysis
OHIP-7T: Oral Health Impact Profile the Taiwanese (Chinese) version
ES: Cohen’s standardized effect size
ANOVA: One-way analysis of variance
NAS: The satisfaction cluster was the lowest satisfaction score at every dimension
SDG: The satisfaction cluster was the lowest satisfaction score at every dimension, but a slightly higher satisfied score
AS: The satisfaction cluster of lower satisfaction pattern was found in the dimension of chewing ability, stability, doctor and general
QS: The satisfaction cluster is quite satisfied at most dimensions except for chewing ability and stability
HS: The satisfaction cluster of higher satisfaction score in six dimensions;
CI: Confidence interval
OR: Odds ratio
